# Temporal Variations in Patterns of *Clostridioides difficile* Strain Diversity and Antibiotic Resistance in Thailand

**DOI:** 10.3390/antibiotics10060714

**Published:** 2021-06-13

**Authors:** Supapit Wongkuna, Tavan Janvilisri, Matthew Phanchana, Phurt Harnvoravongchai, Amornrat Aroonnual, Sathid Aimjongjun, Natamon Malaisri, Surang Chankhamhaengdecha

**Affiliations:** 1 Department of Biochemistry, Faculty of Science, Mahidol University, Bangkok 10400, Thailand; supapit.won@gmail.com (S.W.); tavan.jan@mahidol.ac.th (T.J.); 2 Department of Molecular Tropical Medicine and Genetics, Faculty of Tropical Medicine, Mahidol University, Bangkok 10400, Thailand; matthew.pha@mahidol.edu; 3 Department of Biology, Faculty of Science, Mahidol University, Bangkok 10400, Thailand; phurt.har@mahidol.edu (P.H.); nattamon.nut.ma@gmail.com (N.M.); 4 Department of Tropical Nutrition and Food Science, Faculty of Tropical Medicine, Mahidol University, Bangkok 10400, Thailand; amornrat.aro@mahidol.edu; 5 Graduate Program in Molecular Medicine, Faculty of Science, Mahidol University, Bangkok 10400, Thailand; sathid.ex@gmail.com

**Keywords:** *C. difficile* infection, molecular analysis, toxin production, antibiotic resistance

## Abstract

*Clostridioides difficile* has been recognized as a life-threatening pathogen that causes enteric diseases, including antibiotic-associated diarrhea and pseudomembranous colitis. The severity of *C. difficile* infection (CDI) correlates with toxin production and antibiotic resistance of *C. difficile.* In Thailand, the data addressing ribotypes, toxigenic, and antimicrobial susceptibility profiles of this pathogen are scarce and some of these data sets are limited. In this study, two groups of *C. difficile* isolates in Thailand, including 50 isolates collected from 2006 to 2009 (THA group) and 26 isolates collected from 2010 to 2012 (THB group), were compared for toxin genes and ribotyping profiles. The production of toxins A and B were determined on the basis of toxin gene profiles. In addition, minimum inhibitory concentration of eight antibiotics were examined for all 76 *C. difficile* isolates. The isolates of the THA group were categorized into 27 *A^−^B^+^CDT^−^* (54%) and 23 *A^-^B^-^CDT^-^* (46%), while the THB isolates were classified into five toxigenic profiles, including six *A^+^B^+^CDT^+^* (23%), two *A^+^B^+^CDT^−^* (8%), five *A^−^B^+^CDT^+^* (19%), seven *A^−^B^+^CDT^−^* (27%), and six *A^−^B^−^CDT^−^* (23%). By visually comparing them to the references, only five ribotypes were identified among THA isolates, while 15 ribotypes were identified within THB isolates. Ribotype 017 was the most common in both groups. Interestingly, 18 unknown ribotyping patterns were identified. Among eight *tcdA*-positive isolates, three isolates showed significantly greater levels of toxin A than the reference strain. The levels of toxin B in 3 of 47 *tcdB*-positive isolates were significantly higher than that of the reference strain. Based on the antimicrobial susceptibility test, metronidazole showed potent efficiency against most isolates in both groups. However, high MIC values of cefoxitin (MICs 256 μg/mL) and chloramphenicol (MICs ≥ 64 μg/mL) were observed with most of the isolates. The other five antibiotics exhibited diverse MIC values among two groups of isolates. This work provides evidence of temporal changes in both *C. difficile* strains and patterns of antimicrobial resistance in Thailand.

## 1. Introduction

*Clostridioides difficile* (formerly *Clostridium difficile*), belonging to the family *Clostridiaceae* and genus *Clostridioides*, is an obligate anaerobic, Gram-positive, spore-forming, toxin-producing bacillus [1,2]. This organism is well known to cause infectious diarrhea in humans, ranging from mild diarrhea to severe pseudomembranous colitis [3]. *C. difficile* infection (CDI) has been primarily a healthcare-associated illness, which can occur during antibiotic treatment. Furthermore, the ability of *C. difficile* to form spores leads to the problem of recurring infection. The persistence of spores in the physical environment facilitates its transmission [4]. The pathogenesis of CDI is attributed to the production of two major toxins: toxins A and B. Toxin A is an enterotoxin encoded by *tcdA*, and toxin B is a cytotoxin encoded by *tcdB*. Both toxins belong to the family of large clostridial toxins (LCTs) and are located within a 19.6 kb pathogenicity locus (PaLoc) [5]. In addition to toxins A and B, some strains of *C. difficile* also produce a binary toxin (CDT) encoded by two genes, *cdtA* and *cdtB,* on CdtLoc, a separate pathogenicity island [6]. Although CDTs are not directly required for diseases, they have been known to promote the virulence of *C. difficile* by impairing host immunity and acting in synergy with toxins A and B, exacerbating toxicity [7].

Over the recent decades, the epidemiology of CDI has dramatically changed. The epidemiology in North America and Europe and some parts of Asia is well-documented. While ribotype 027 causes major outbreaks in North America and Europe, ribotype 017 is the most dominant ribotype in Asia [8,9]. In Thailand, tcdA-negative, tcdB-positive ribotype 017 is the most prevalent *C. difficile* strain [10,11,12]. However, the occurrence of *C. difficile* has not been studied in all regions of Thailand. Recently, the diversity and prevalence of *C. difficile* have increased and influenced the incidence of CDI in many areas. Several ribotypes have emerged and lead to epidemic infections across the world; for example, ribotype 014/20 in Australia [13,14], ribotype 369 in Japan [15], and ribotype 078 in China [16].

Antibiotic use plays a major role in the development of CDI and recurrent diseases by disrupting the normal flora in the gut and allowing the invasion of *C. difficile* [17,18]. The first-line treatment for CDI is the use of antibiotics, including vancomycin, fidaxomicin, and metronidazole [19,20]. However, drug resistance has become one contributing factor that drives the global prevalence of CDI [21,22,23,24]. Although information on *C. difficile* has been globally expanded, little knowledge of antibiotic susceptibility of *C. difficile* in Thailand is available. Previous studies showed that *C. difficile* isolates in Thailand were susceptible to vancomycin and metronidazole. However, a high resistance level against multiple antibiotics, such as clindamycin, erythromycin, and moxifloxacin has been reported [25,26]. This study was conducted to compare two groups of *C. difficile* clinical isolates collected in different time periods from a University-affiliated tertiary hospital and the National Institute of Health of Thailand. To describe the diversity of *C. difficile* clinical isolates during 2006–2009 and 2010–2012, the presence of toxin genes and ribotype, including toxin levels and antimicrobial susceptibility patterns, were characterized using molecular techniques.

## 2. Results

### 2.1. Toxin Gene Profiles of C. difficile Isolates

**Figure 1:**
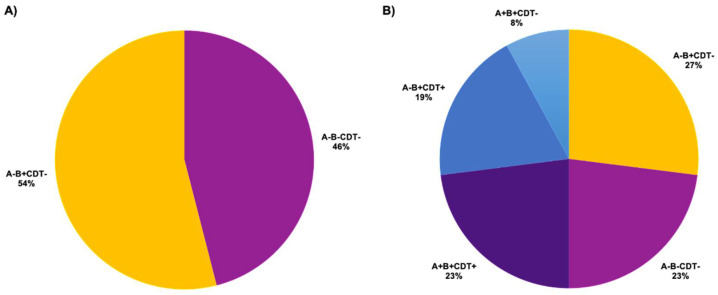
Toxin gene analysis of *C. difficile* isolates. The distribution of toxin profiles in *C. difficile* isolates from (**A**) THA group, which contained *C. difficile* isolates collected from 2006 to 2009 (n = 50) and (**B**) THB group, which contained *C. difficile* isolates collected from 2010 to 2012 (n = 26). Toxin profiles were characterized based on the presence of toxin genes and the deletion of *tcdA* 3′-end. A, B, and CDT represent *tcdA*, *tcdB*, and *cdtAB*.

The multiplex PCR was employed to identify the toxin gene profiles of *C. difficile* isolates. Seventy-six *C. difficile* isolates were classified into five profiles based on the presence of toxin genes. Only two toxigenic types were observed in the THA group. Twenty-seven THA isolates (54%) were characterized as *A^−^B^+^CDT^−^* (toxigenic), and 23 THA isolates (46%) were *A^−^B^−^CDT^−^* (non-toxigenic) (Figure 1A). All 27 isolates in the THA group that were previously positive for *tcdA* carried the *tcdA* 3′-end deletion (Supplementary Table S1). Later, they were grouped as *tcdA*-negative isolates instead. Thus, none of the toxigenic isolates in the THA group were *tcdA*-positive. In the THB group, six isolates (23%) were classified as *A^+^B^+^CDT^+^*, five isolates (19%) as *A^−^B^+^CDT^+^*, two isolates (8%) as *A^+^B^+^CDT^−^*, seven isolates (27%) as *A^−^B^+^CDT^−^*, and six isolates (23%) as *A^−^B^−^CDT^−^* (Figure 1B). Among *tcdA*-negative isolates in the THB group, in 12 isolates (63%) were found the deletion regions within the 3′-end (Table S1). Based on the molecular analysis, around 54% of the THA isolates and 77% of the THB isolates were toxigenic (Figure 1). The most dominant toxigenic type was *A^−^B^+^*, which was about 54% of THA isolates and 46% of THB isolates.

### 2.2. Ribotypes of C. difficile Isolates

**Figure 2:**
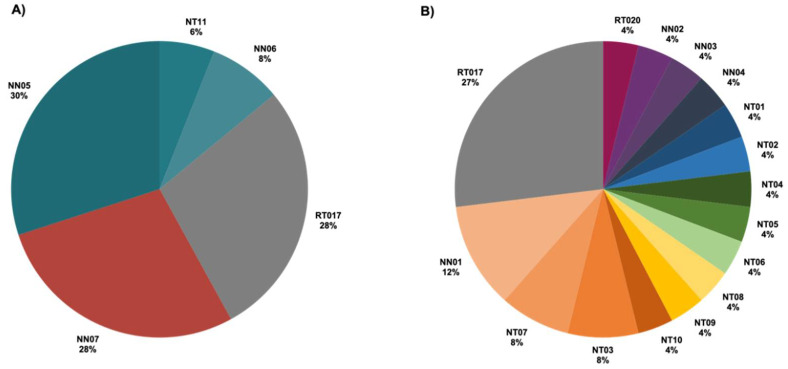
Ribotypes of *C. difficile* isolates using PCR ribotyping method. (**A**) The distribution of ribotypes among *C. difficile* isolates in the THA group (n = 50). (**B**) The distribution of ribotypes among *C. difficile* isolates in the THB group (n = 26). PCR ribotyping was performed on 16S and 23S rRNA genes. Ribotypes were assigned based on the band patterns of gel electrophoresis. RT017 and RT020 represent the standard *C. difficile* ribotypes. NT and NN represent new ribotype patterns of *C. difficile* toxigenic and non-toxigenic isolates, respectively.

The band patterns of 16S and 23S rRNA PCR products were compared to the reference *C. difficile* ribotypes (Figure S1). Based on PCR ribotyping, THA isolates were separated into five ribotypes (Figure 2A). Ribotype 017 was the only standard ribotype found in the THA group, whereas the other four ribotypes showed different patterns from the standards (NN or NT). The dominant ribotype was NN05, followed by ribotype 017 and NN07. Even though the number of isolates in the THB group was lower compared to the THA group, THB isolates were classified into 15 ribotypes (Figure 2B). Ribotype 017 had the highest prevalence in the THB group with seven isolates (27%). Only one isolate (4%) was classified as ribotype 020. Alternatively, the other 14 isolates in the THB group showing distinct ribotyping patterns compared to the references were classified into 13 unknown ribotypes. The distribution of toxin gene profiles and ribotyping profiles is elaborated in Table 1. Diverse ribotypes were observed with each toxin gene profile; for instance, the *A^+^B^+^CDT^+^* group was composed of five ribotyping patterns, RT020, NT01, NT03, NT05, and NT06 (Table 1). These results suggest a high diversity of *C. difficile* isolates in Thailand.

### 2.3. Toxin Production of C. difficile Isolates

**Figure 3:**
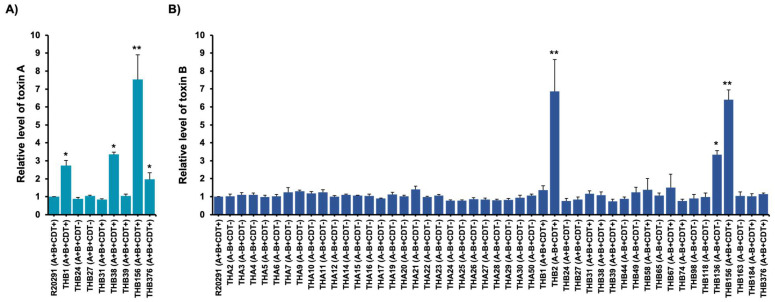
The relative level of toxin A and B production of *C. difficile* isolates. Toxigenic *C. difficile* isolates in the THA group; *B^+^* (n = 27) and the THB group; *A^+^* (n = 8) and *B^+^* (n = 20) were subjected to indirect ELISA. (**A**) The light blue bars represent toxin A, and (**B**) the dark blue bars represent toxin B. The graph shows average values of 3 independent samples in the experiments. Error bars refer to mean ± SEM, *p* < 0.05, *, and *p* < 0.01, **.

Toxin production of *C. difficile* is a significant factor causing CDI [27]. In this study, toxin production of toxigenic *C. difficile* isolates, including *A^+^B^+^*, *A^+^B^−^*, and *A^−^B^+^*, was accessed using indirect ELISA. The toxin levels of individual isolates were compared to the toxin production of *C. difficile* R20291 (*A^+^B^+^CDT^+^*), a recent emergence of a highly virulent bacterium. The unique ability of hypervirulent strain R20291 is associated with an increase in toxin production [28]. The amounts of toxins A and B were similar among toxigenic isolates in the THA and THB groups. Notably, four toxin-positive isolates, THB1, THB38, THB156, and THB376, significantly increased toxin A levels (2–9 folds) compared to R020291 (Figure 3A). Toxigenic THA isolates were found to produce similar levels of toxin B to the reference strain. Three isolates, THB2, THB136, and THB156, significantly produced greater levels of toxin B (3–6 folds) compared to the reference strain (Figure 3B). Interestingly, THB156 was the only toxigenic isolate that produced a significantly high level of toxin A and B. On the basis of these results, many THB isolates represented high toxin producers, suggesting increased toxin production of toxigenic *C. difficile* isolates in Thailand.

### 2.4. Antimicrobial Resistance Profiles of C. difficile Isolates

**Figure 4:**
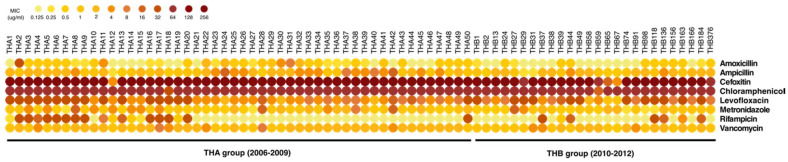
The MIC values of *C. difficile* isolates in Thailand. Eight antibiotics were used to investigate MIC values of *C. difficile* isolates in THA group (n = 50) and THB group (n = 26) using broth dilution method. The colors represent different MIC values.

Antibiotic resistance has become one of the major challenges of CDI treatment. In this study, the antimicrobial susceptibility of the 76 *C. difficile* isolates was determined using the minimum inhibitory concentration (MIC) method. A variety of MIC values of eight antibiotics were observed across *C. difficile* isolates (Figure 4). Antibiotic susceptibility patterns of two groups of isolates are summarized in Table 2. In the THA group, 48 isolates (96%) were susceptible to amoxicillin with an MIC_90_ of 2 μg/mL, while 46 isolates (92%) were susceptible to ampicillin with an MIC_90_ of 4 μg/mL. All THA isolates were resistant to chloramphenicol with an MIC_90_ of ≥ 64 μg/mL. In addition, all THA isolates were resistant to cefoxitin, except one isolate with an MIC_90_ of 256 μg/mL. Conversely, all isolates in the THA group were susceptible to metronidazole with an MIC_90_ of 4 μg/mL. Amoxicillin and ampicillin showed potent activity against all THB isolates with an MIC_90_ of 2 and 4 μg/mL, respectively. Additionally, most THB isolates were resistant to chloramphenicol with an MIC_90_ of ≥ 64 μg/mL (96.15%), followed by cefoxitin with an MIC_90_ of 256 μg/mL (92.31%). None of the isolates in the THB group were resistant to metronidazole and only three THB isolates (11.54%) were resistance to vancomycin. In addition, three (11.54%) and two (7.69%) of THB isolates were resistant to levofloxacin and rifampicin, respectively. Minor differences in the MIC range between THA and THB isolates were observed (Table 3). For instance, chloramphenicol showed an MIC range of 32- ≥ 64 μg/mL in THA isolates, and 16- ≥ 64 μg/mL in THB isolates. A slightly greater ratio of resistant isolates was shown in THA isolates compared to THB isolates. Overall, two groups of isolates showed similar patterns of MIC values. Most THA and THB isolates were susceptible to all antibiotics, except cefoxitin and chloramphenicol, which showed the highest MIC ranges and resistance rates (Table 2). In total, 49 (98%) of the THA isolates and 23 (88.46%) of the THB isolates were resistant to more than one antibiotic. Most of them were resistant to chloramphenicol, cefoxitin, and levofloxacin, which belong to different antibiotic classes. These findings demonstrated multidrug-resistant (MDR) strains among Thai *C. difficile* isolates (Table S2).

## 3. Discussion

*C. difficile* infection (CDI) has occurred worldwide over recent decades. The prevalence and epidemiology of *C. difficile* in many regions are well documented [31,32]. However, information on *C. difficile* occurrences in Thailand remains limited. This work was conducted to continuously update information on *C. difficile* clinical isolates in Thailand by comparing two groups of clinical isolates that were collected in different time periods. *C. difficile* isolates were classified based on molecular features, including toxin genes and the 16S–23S rRNA intergenic spacer regions [33,34]. Normally, three major toxigenic types (*A^+^B^+^*, *A^+^B^−^*, *A^−^B^+^*) cause clinical incidences of CDI. The toxigenic type *A^+^B^+^* is the most common among toxigenic types [35,36]. However, the presence of *tcdA* 3′-end deletion has been detected in many clinical isolates, resulting in toxin A-negative *C. difficile* isolates [37,38]. About half of *C. difficile* isolates collected during 2006–2009 (THA group) were toxigenic with the highest occurrence of *A^-^B^+^* isolates (Figure 1A). Although isolates used in this study were obtained from the patients with CDI, consistent with previous studies, non-toxigenic strains were highly detected from clinical samples due to the mix of both the non-toxigenic and toxigenic populations and isolation method [10,39,40]. The population sizes of non-toxigenic and toxigenic *C. difficile* isolates in Thailand during 2006–2018 were comparable. The most dominant toxigenic isolates were tcdA-negative and tcdB-positive (*A^−^B^+^*) [10]. In contrast, the majority of *C. difficile* isolates collected during 2010–2012 (THB group) were toxigenic, and toxin gene profiles increased to five types, *A^−^B^−^CDT^−^*, *A^−^B^+^CDT^−^*, *A^+^B^+^CDT^−^*, *A^−^B^+^CDT^+^*, and *A^+^B^+^CDT^+^* (Figure 1B). However, no *A^−^B^−^CDT^+^* was detected in this study, corresponding to the previous study showing low prevalence of binary toxin-positive but toxin A- and B-negative *C. difficile* strains in France [41]. Some *C. difficile* isolates have the binary toxin gene (*CDT*), an actin-specific ADP-ribosyl transferase encoded by two genes, *cdtA* and *cdtB* on the CDT locus (CdtLoc) [6,42]. The binary toxins are widely observed in hypervirulent *C. difficile,* such as the ribotypes 027 and 078, which cause higher severity of CDI [43,44]. Therefore, the binary toxin may serve as an additional virulent factor by enhancing the production of toxins A and B. Our findings indicate a higher prevalence of toxigenic isolates in Thailand from 2010 to 2012.

Currently, PCR ribotyping is a general technique for epidemiological distinction of *C. difficile* isolates. This method amplifies polymorphic sequences between 16S and 23S intergenic spacer regions, which vary among strains [33,45,46]. It is the most common method employed for molecular analysis of *C. difficile* strains and is considered the gold standard method for *C. difficile* typing [10,11,33,47]. A similar incidence shown in the analysis of toxin genes was also observed with the ribotyping profiles. The number of ribotypes found during 2010–2012 was up to 16 ribotypes, from five ribotypes identified during 2006–2009 (Figure 2). *C. difficile* ribotype 017 has been recognized as a major cause of CDI outbreaks in Asia, and ribotype 020 is also a common strain [12,48]. Ribotype 017 was also the most frequently found in Thailand [11]. Consistent with this study, the most common ribotype in both groups was ribotypes 017. Besides, there were unknown ribotypes which showed different amplified patterns compared to the references between the two groups. However, we could not compare the PCR ribotyping patterns of the unknown ribotypes to other unknown ribotypes discovered in the previous studies in Thailand due to the limitation of this method. Other techniques, including pulse-field gel electrophoresis (PFGE), restriction endonuclease analysis (REA), and multilocus variable-number tandem-repeat analysis (MLVA), can be applied to improve typing of *C. difficile* strains [49,50]. Based on PCR ribotyping, molecular epidemiology of *C. difficile* isolated in Thailand significantly differs from other regions where ribotypes 027, 014/20, 002, 106, and 001 have dominated in North America and ribotypes 027, 014, 001, and 078 have frequently been isolated in Europe [51,52]. On the basis of toxin genes and ribotype identification, the diversity of *C. difficile* isolates in Thailand has increased over time.

Toxins A and B are the primary virulence factors contributing to the pathogenesis of CDI. They are considered to cause severe diseases [53]. Several studies have revealed that *A^−^B^+^* strains can cause the same range of disease as isolates producing both, but a few pathogenic isolates have been found as *A^+^B^−^* [54,55,56]. In the current study, none of the toxigenic isolates were classified as *A^+^B^−^*, supporting the finding that toxin B is important for the pathogenesis of *C. difficile* without the presence of toxin A. This implied that pathogenic *C. difficile* isolates in Thailand were mainly influenced by the production of toxin B. Based on the relative quantification of toxins in this study, three of eight *tcdA*-positive (*A^+^*) isolates showed significantly greater production of toxin A compared to a recent hypervirulent *C. difficile* strain. Most *tcdB*-positive (*B^+^*) isolates produced toxin B at the same level as the reference strain, of which only three *tcdB*-positive isolates in the THB group significantly increased the level of toxin B (Figure 3). Remarkably, most isolates that produced high levels of toxins A and B were binary toxin-positive (*CDT^+^*) isolates. A high toxin production is one of the features of hypervirulent strains associated with severity of disease [57,58,59]. Markedly, an increase in toxin production is influenced by binary toxins [6,60]. Therefore, the higher amount of toxins produced by isolates in this study might be associated with the presence of binary toxin genes.

Antibiotic resistance has become one of the most important virulence factors associated with the development of CDI. The expansion of strain diversity advocates antibiotic resistance in *C. difficile* [24,61,62]. To determine the direction of the antibiotic susceptibility of Thai *C. difficile* isolates, two groups of isolates were tested against several classes of antibiotics, which are recommended in infectious diarrhea [63,64]. None of the isolates fully resisted metronidazole, but three isolates showed intermediate resistance. However, 9 of 76 isolates had full resistance to vancomycin. This incidence was also observed in several studies with reduced susceptibility to vancomycin [22,65]. Our observations suggest a high efficiency of metronidazole for treating CDI, that also relates to the previous studies in Thailand [25,26]. Beta-lactam groups of antibiotics are most frequently correlated with CDI [66]. Several studies reported a low level of resistance to this antibiotic group [61,62]. In this study, amoxicillin and ampicillin also showed potent action against *C. difficile* isolates in Thailand. This supported the fact that antibiotics in the same class provide equal efficacy. Nevertheless, fluoroquinolones (ciprofloxacin, levofloxacin, moxifloxacin, norfloxacin) and cephalosporins (cefazolin, cefepime, ceftazidime, ceftriaxone, cefuroxime, cefotetan, cefoxitin) are common antibiotic groups used for treating bacterial infection in the clinical setting, and they continue to promote CDI [67,68]. The same incidence was detected in this study, in which the majority of *C. difficile* isolates were resistant to levofloxacin and cefoxitin. Resistance to chloramphenicol is rare in *C. difficile*. Only a small number of isolates have been reported to be chloramphenicol resistant [24,69]. Contrary to our observations, all isolates fully resisted chloramphenicol, except for one that showed intermediate resistance. In addition, the reduced susceptibility to rifampicin in *C. difficile* clinical strains has been reported in Asia, Europe, and North America [69,70,71]. Correspondingly, rifampicin-resistant isolates were detected in the current study. On the basis of antibiotic resistance analysis, most *C. difficile* isolates in this study were resistant to multiple antibiotics, increasing the chance of treatment failure. Although *C. difficile* isolates between two periods showed distinct diversity, the difference in the patterns of antibiotic resistance was not observed in this study.

In summary, *C. difficile* isolates from patients diagnosed with diarrhea during 2006–2009 and 2010–2012 were characterized for toxigenic types, ribotypes, toxin production, and antibiotic resistance. The toxigenic profiles found in Thailand rose to five types, including *A^−^B^+^CDT^−^, A^+^B^+^CDT^+^*, *A^+^B^+^CDT^−^*, *A^−^B^+^CDT^+^*, and *A^−^B^−^CDT^−^*. In particular, ribotype 017 was predominant among clinical isolates in Thailand. Additionally, 18 unknown ribotypes were discovered in Thai isolates. Some *C. difficile* isolates in Thailand were able to produce similar levels of toxins A and B to the toxins of the hypervirulent *C. difficile* strain, R20291. There was no difference in susceptibility to vancomycin and metronidazole between two periods, supporting the fact that they are primary antibiotics for CDI therapy. In addition, amoxicillin, ampicillin, and rifampicin also had an effective impact on treating isolates in Thailand. Based on these findings, this study presents temporal changes in *C. difficile* strain diversity and patterns of antimicrobial resistance in Thailand, which will be useful for surveillance.

## 4. Materials and Methods

### 4.1. Sample Collection and Bacterial Culture

In total, 76 *C. difficile* clinical isolates were obtained from a University-affiliated tertiary hospital and the National Institute of Health of Thailand. The isolation of *C. difficile* from stool samples of diarrheal patients was performed in previous studies [39,40]. These isolates were separated into 2 groups based on collection periods. The THA group was composed of 50 isolates collected from 2006 to 2009, and the THB group contained 26 isolates collected from 2010 to 2012. Each isolate was cultured on cycloserine–cefoxitin fructose agar (CCFA) for 24 h at 37 °C under anaerobic conditions (Coy Laboratory Products, Glass Lake, MI, USA) supplemented with 0.1% taurocholate to recover and enrich *C. difficile* cells. A single colony was cultured in fresh brain heart infusion (BHI) broth and incubated in an anaerobic chamber at 37 °C for 24–48 h. The culture was preserved with 10% (*v*/*v*) glycerol at −80 °C for further use.

### 4.2. Toxin Genotyping

Genomic DNA of *C. difficile* isolates was extracted from BHI culture using an E.Z.N.A.^®^ Stool DNA kit (Omega Bio-tek, Norcross, GA, USA), according to the manufacturer’s instructions. DNA purity and concentration were assessed by NanoDrop^TM^ spectrophotometer (Thermo Fisher Scientific, Waltham, MA, USA). Toxigenic profiles of all *C. difficile* isolates were analyzed by multiplex PCR with 5 specific primer pairs, *tcdA, tcdB, cdtA, cdtB*, and 16S rDNA (Table 3). The PCR reaction was conducted in a total volume of 20 µL containing 25–200 ng of genomic DNA, 0.8 mM dNTPs, 5 mM MgCl_2_, 1× PCR buffer, (500 mM KCl, 100 mM tris-HCl, pH 9.1), 1U *Taq* DNA polymerase (Vivantis, kuala Lumpur, Malaysia), and 0.2 µM primers. Amplification was performed under a thermal cycler with cycling conditions including a predenaturation at 92 °C for 5 min, 30 cycles of denaturation at 92 °C for 20s, an annealing at 58 °C for 65s, and an extension at 68 °C for 90s, and a final extension at 60 °C for 5 min.

In addition, the deletion in repeating regions at the 3′ end of the *tcdA* gene was investigated using the NK9 and NKV011 primers (Table 3) by Kato et al. 1999 [72]. PCR reaction was performed under the same conditions of the multiplex PCR. The thermocycler conditions included a predenaturation at 94 °C for 6 min, followed by 37 cycles of denaturation at 94 °C for 20 s, an annealing at 55 °C for 30 s, and an extension at 60 °C for 120 s, and a final extension at 60 °C for 10 min. The PCR products were visualized using electrophoresis with 1.2% agarose gel and strained with ethidium bromide.

### 4.3. PCR Ribotyping

PCR ribotyping was performed based on the 16S–23S rRNA intergenic spacer regions described by Bidet et al. 1999 [73]. The primer sequences were 5′-GTGCGGCTGGATCACCTCCT-3′ (16S primer) and 5′-CCCTGCACCCTTATTACCTTGACC-3′ (23S primer). The PCR reaction was conducted in a total volume of 20 µL composed of 25–200 ng genomic DNA, 0.2 mM dNTPs, 0.2 µM primers, 1.5 mM MgCl_2_, 10× PCR buffer (500 mM KCl, 100 mM Tris-HCl, pH 9.1), 1U *Tag* DNA polymerase, and deionized water. The thermocycler profile consisted of an initial denaturation at 95 °C for 5 min, followed by 35 cycles at 95 °C for 1 min, 57 °C for 1 min, and 72 °C for 1 min, and a final extension at 72 °C for 10 min. Amplification products were separated by electrophoresis in 3% agarose gel for 6 h with 85 V in 1× Tris-borate EDAT (TBE) buffer. The electrophoresis patterns were visualized under UV light after staining with ethidium bromide. The high-resolution image was captured and analyzed with a gel documentation system. The resulting band patterns were visually compared to PCR ribotypes of the reference strains, *C. difficile* PCR ribotypes 001, 012, 017, 020, 023, 027, 046, 056, 077, 081, 095, 106, and 117.

### 4.4. Quantification of Toxins A and B

Toxigenic *C. difficile* isolates (n = 47) and the reference strain, *C. difficile* R20291, were inoculated on CCFA agar plates. A single colony was cultured in fresh BHI media. A total of 1% of bacterial culture was sub-cultured into fresh BHI media for 48 h at 37 °C. The supernatant was collected from the culture using centrifugation at 5000× *g* for 10 min and sterilized by passing through a 0.22 µm membrane. Total protein was measured using Bradford’s assay (Clive G et al., 1989). Indirect enzyme-link immunosorbent assay (ELISA) was performed to quantify the level of toxins A and B. Initially, 96-well polystyrene microtiter plates were coated with 100 μL of 5 mg/mL supernatant in 0.5 M carbonate buffer (pH 9.4) and incubated overnight at 4 °C. The plates were washed three times with 200 μL of 1× PBS. Then, 200 μL of blocking solution (1% BSA) was added to wells. The plates were incubated for 1 h at room temperature and washed with PBS-T (0.05% Tween-20, pH 7.4). The 100 μL final 1:500 dilution of mouse anti-toxin A (Abcam, Cambridge, UK) or 1:250 dilution of mouse anti-toxin B (Bio-Rad, Hercules, CA, USA) was added to wells. The plates were incubated for 1 h at 37 °C and washed three times with 100 µL of PBS-T at room temperature. Finally, 50 µL of 1:4 dilution of Equilibrate SignalStain^®^ Boost IHC Detection Reagent (HRP, anti-mouse) (Cell Signaling, Beverly, MA, USA) was added to wells. The plates were then incubated for 1 h at 37 °C and washed three times with 1× PBS. Finally, 100 μL TMB (3,3′,5,5′-tetramethylbenzidine) substrate (Seracare, Milford, MA, USA) was added to wells. After 10 min of incubation at 37 °C, the reaction was stopped by addition of 100 μL of 2 N hydrochloric acid. The absorbance at 450 nm was measured by microplate reader (Tecan, Switzerland). The relative levels of toxin production were compared to the reference strain, *C. difficile* R20291.

### 4.5. Minimal Inhibitory Concentration (MIC) Testing

The minimal inhibitory concentration (MIC) testing was performed using 96-well broth dilution in triplicate. Nine antibiotics, including metronidazole (0.0625–16 μg/mL), vancomycin (0.0625–16 μg/mL), amoxicillin (0.125–32 μg/mL), ampicillin (0.125–32 μg/mL), cefoxitin (2–256 μg/mL), chloramphenicol (0.25–32 μg/mL), levofloxacin (0.125–32 μg/mL), and rifampicin (0.125–32 μg/mL) were subjected to MIC testing. A single colony of *C. difficile* on CCFA was inoculated into fresh BHI medium. After overnight incubation, *C. difficile* culture was transferred to freshly prepared Wilkins-Chalgren broth until the OD_600_ reached 0.6 (~10^8^ CFU/mL). Two-fold serial dilutions of antibiotics (0.125–512 μg/mL) were prepared in a 96-well plate at a total volume of 200 μL. A total of 10 μL of bacterial suspension (~10^6^ CFU/mL) was then inoculated into antibiotic plates. The 96-well microplates were incubated at 37 °C under anaerobic conditions for 48 h. The OD_600_ at the end point was measured using a spectrophotometer. The MIC values were defined as the lowest concentration of antibiotic where no growth of bacteria was observed. The MIC results were categorized according to the guidelines of the Clinical and Laboratory Standards Institute (CLSI), http://www.clsi.org/ accessed on 1 June 2021; the European Committee on Antimicrobial Susceptibility Testing (EUCAST), http://www.eucast.org/ accessed on 1 June 2021; and published data [29,30].

## Figures and Tables

**Table 1:** Summary of toxin gene profiles and ribotyping profiles of 76 *C. difficile* isolates in Thailand.

Toxigenic Profile	No. of Isolates		No. of Isolates of																			
	THA (n = 50)	THB (n = 26)	RT017	RT020	NN01	NN02	NN03	NN04	NN05	NN06	NN07	NT01	NT02	NT03	NT04	NT05	NT06	NT07	NT08	NT09	NT10	NT11
*A^+^B^+^CDT^+^*		6		1								1		2		1	1					
*A^+^B^+^CDT^-^*		2																2				
*A^-^B^+^CDT^+^*		5	1										1		1				1		1	
*A^-^B^+^CDT^-^*	27	7	20						9	1										1		3
*A^-^B^-^CDT^-^*	23	6			3	1	1	1	6	3	14											

**Table 2:** Antibiotic susceptibility patterns of *C. difficile* isolates in Thailand.

Antibiotics	MIC Range (µg/mL)		MIC_50_ (µg/mL)		MIC_90_ (µg/mL)		Breakpoints (µg/mL)	Susceptible (%)		Intermediate (%)		Resistant (%)	
	THA (n = 50)	THB (n = 26)	THA	THB	THA	THB	S/I/R	THA	THB	THA	THB	THA	THB
Amoxicillin	≤0.125-32	≤0.125-0.5	0.5	≤0.125	2	0.5	≤4/8/≥ 16 ≤2/4/≥ 8	96	100	2	0	2	0
Ampicillin	0.25-16	0.25-4	2	2	4	2	≤0.5/1/≥ 2	92	100	4	0	4	0
Cefoxitin	4-256	4-256	256	256	256	256	≤16/32/≥ 64	2	7.69	0	0	98	92.31
Chloramphenicol	32- ≥ 64	16- ≥ 64	≥64	≥64	≥64	≥64	≤8/16/≥ 32	0	0	0	3.85	100	96.15
Levofloxacin	2- ≥ 32	1- ≥ 32	8	8	≥32	≥32	-/-/≥8 ^a^	-	-	-	-	56	11.54
Metronidazole	0.25-16	0.25- ≥ 16	1	1	4	2	≤8/16/≥ 32	96	96.15	4	3.85	0	0
Rifampicin	≤0.125- ≥ 32	≤0.125- ≥ 32	≤0.125	≤0.125	≥32	≥32	≤0.06/0.012-16/≥ 32 ^b^	0	0	74	92.31	26	7.69
Vancomycin	1-8	0.5-4	2	1	4	4	≤2/-/> 2 ^c^	88	88.46	-	-	12	11.54

* Clinical breakpoints determining the susceptibility categories: S; susceptible, I; intermediate, and R; resistance. All breakpoints were recommended by CLSI, except ^a^ breakpoint for levofloxacin by published data [29], ^b^ breakpoint for rifampicin by published data [30], and ^c^ breakpoint for vancomycin by EUCAST.

**Table 3:** Sequences of primers for amplifying toxin genes of *C. difficile* and amplicon size.

Analysis	Target Gene	Primer Name	Sequence (5′-3′)	Amplicon Size (bp)
Multiplex PCR	*tcdA*	tcdA-F	GTATGGATAGGTGGAGAAGTCAGTG	632
		tcdA-R	CGGTCTAGTCCAATAGAGCTAGGTC	
	*tcdB*	tcdB-F	GAAGATTTAGGAAATGAAGAAGGTGA	441
		tcdB-R	AACCACTATATTCAACTGCTTGTCC	
	*cdtA*	cdtA-F	ATGCACAAGACTTACAAAGCTATAGTG	260
		cdtA-R	CGAGAATTTGCTTCTATTTGATAATC	
	*cdtB*	cdtB-F	ATTGGCAATAATCTATCTCCTGGA	179
		cdtB-R	CTTGTTCTGGTACCAAATAATCCG	
	*16S rRNA*	UFU-L	GCCTAACACATGCAAGTCGA	800
		802-R	TACCAGGGTATCTAATCC	
*tcdA* 3′-end deletion	*tcdA*	NK9	CCACCAGCTGCAGCCATA	2535
		NKV011	TTTTGATCCTATAGAATYTAACTTAGTAAC	

## Data Availability

Data are contained within the article.

## Supplementary Materials

The following are available online at https://www.mdpi.com/article/10.3390/antibiotics10060714/s1, Figure S1: PCR ribotyping of (A) standard *C. difficile* strains and (B) *C. difficile* isolates in this study, Table S1: Toxigenic profiles and ribotype of 76 *C. difficile* clinical isolates from Thailand, Table S2: Antimicrobial susceptibility of 76 *C. difficile* clinical isolates from Thailand against 8 antibiotics.
