# Supplementary material for: Temporal Variations in Patterns of Clostridioides difficile Strain Diversity and Antibiotic Resistance in Thailand
Source: Antibiotics (Basel). 2021 Jun 13;10(6):714. doi: 10.3390/antibiotics10060714 (PMC8231780; doi:10.3390/antibiotics10060714)
Supplement: Supplementary file 1 [file antibiotics-10-00714-s001.zip › antibiotics-1239851-SI/Suplementary Figure and Tables/FigureS1.pdf]

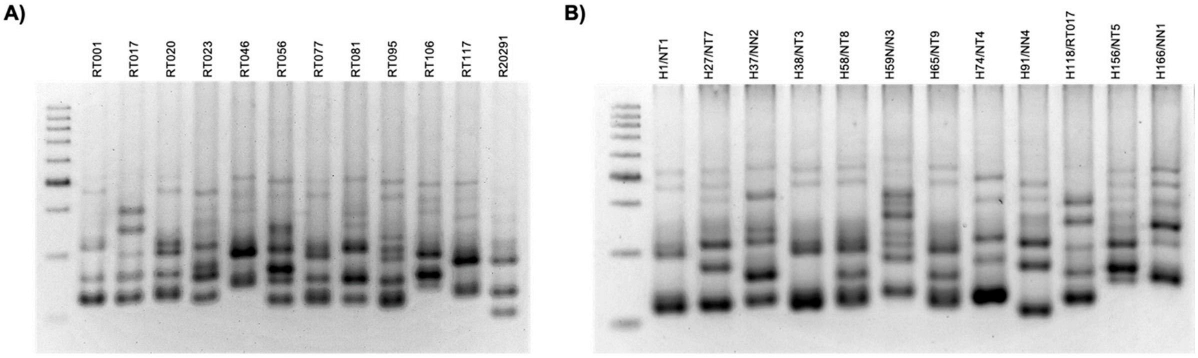

Figure S1. PCR ribotyping of A) standard C. difficile strains and B) C. difficile isolates in this study. The PCR was performed using 16S and 23S rRNA primer to amplify the intergenic spacer regions. The amplified products were visual using gel electrophoresis.
